# Synthetic lethality of *Mycobacterium tuberculosis* NADH dehydrogenases is due to impaired NADH oxidation

**DOI:** 10.1128/mbio.01045-23

**Published:** 2023-11-30

**Authors:** Yuanyuan Xu, Sabine Ehrt, Dirk Schnappinger, Tiago Beites

**Affiliations:** 1 Department of Microbiology and Immunology, Weill Cornell Medical College, New York, USA; Washington University School of Medicine, St. Louis, Missouri, USA

**Keywords:** *Mycobacterium tuberculosis*, antimicrobial activity, pathogenesis, respiration, nicotinamide adenine dinucleotide

## Abstract

Type 2 NADH dehydrogenase (Ndh-2) is an oxidative phosphorylation enzyme discussed as a promising drug target in different pathogens, including *Plasmodium falciparum* and *Mycobacterium tuberculosis* (*Mtb*). To kill *Mtb*, Ndh-2 needs to be inactivated together with the alternative enzyme type 1 NADH dehydrogenase (Ndh-1), but the mechanism of this synthetic lethality remained unknown. Here, we provide insights into the biology of NADH dehydrogenases and a mechanistic explanation for Ndh-1 and Ndh-2 synthetic lethality in *Mtb*. NADH dehydrogenases have two main functions: maintaining an appropriate NADH/NAD+ ratio by converting NADH into NAD+ and providing electrons to the respiratory chain. Heterologous expression of a water-forming NADH oxidase (Nox), which catalyzes the oxidation of NADH, allows us to distinguish between these two functions and shows that Nox rescues *Mtb* from Ndh-1/Ndh-2 synthetic lethality, indicating that NADH oxidation is the essential function of NADH dehydrogenases for *Mtb* viability. Quantification of intracellular levels of NADH, NAD, ATP, and oxygen consumption revealed that preventing NADH oxidation by Ndh-1/Ndh-2 depletes NAD(H) and inhibits respiration. Finally, we show that Ndh-1/Ndh-2 synthetic lethality can be achieved through chemical inhibition.

**IMPORTANCE:**

In 2022, it was estimated that 10.6 million people fell ill, and 1.6 million people died from tuberculosis (TB). Available treatment is lengthy and requires a multi-drug regimen, which calls for new strategies to cure *Mycobacterium tuberculosis* (*Mtb*) infections more efficiently. We have previously shown that simultaneous inactivation of type 1 (Ndh-1) and type 2 (Ndh-2) NADH dehydrogenases kills *Mtb*. NADH dehydrogenases play two main physiological roles: NADH oxidation and electron entry into the respiratory chain. Here, we show that this bactericidal effect is a consequence of impaired NADH oxidation. Importantly, we demonstrate that Ndh-1/Ndh-2 synthetic lethality can be achieved through simultaneous chemical inhibition, which could be exploited by TB drug development programs.

## INTRODUCTION

Oxidative phosphorylation is an essential cell process for *Mycobacterium tuberculosis* (*Mtb*)—the etiological agent of tuberculosis (TB)—both in replicating and non-replicating conditions (1). This has prompted efforts to identify small molecules that effectively inhibit oxidative phosphorylation, leading to the discovery of the ATP synthase inhibitor bedaquiline (2)—a drug that has contributed to the treatment shortening of drug-resistant TB (3).

Given the success of bedaquiline, TB drug development programs have been exploring other possible drug targets in *Mtb* oxidative phosphorylation. In this context, type 2 NADH dehydrogenase (Ndh-2) has been discussed as a promising target. Indeed, Ndh-2 inhibition has been proposed as an effective strategy to eradicate infections with other pathogens, like *Plasmodium falciparum* (4) (malaria etiological agent) or *Leishmania* sp. (5) (leishmaniasis etiological agent). The *Mtb* genome harbors two genes encoding Ndh-2 enzymes, *ndh* and *ndhA*. Transposon mutant screenings identified *ndh* as required for optimal growth of *Mtb in vitro* (6, 7). Moreover, phenothiazines, which are active against replicating and non-replicating bacilli (1), were shown to inhibit Ndh-2 activity and respiration (8). These data were thus consistent with an essential role for Ndh-2 in *Mtb* oxidative phosphorylation, which together with the absence of a homologous enzyme in humans motivated the identification of specific Ndh-2 inhibitors.

In addition to phenothiazines, which we later showed to inhibit *Mtb in vitro* growth independently of Ndh-2 (9), multiple small molecules have been proposed to specifically inhibit Ndh-2 in *Mtb*: quinolones (10), quinolinyl pyrimidines (11), molecules with thioquinazoline and tetrahydroindazole cores (12), diphenyleneiodonium analogs (13), 2-mercapto-quinazolinones (14), 7-phenyl benzoxaborole compound series (15), and, more recently, tricyclic spirolactams (16). However, the activity of these compounds against *Mtb* was only demonstrated in *in vitro* conditions, and an *Mtb* strain in which both genes encoding Ndh-2 enzymes have been deleted (*Mtb Δndh-2*) is only mildly attenuated in a mouse model of infection, suggesting that inhibition of Ndh-2 alone will not kill *Mtb* during infection (9). We also showed that Ndh-2 activity is conditionally essential for *Mtb in vitro* growth depending on the presence of long-chain fatty acids. Interestingly, a heterologous expression of the water-forming NADH oxidase *nox* was able to rescue *Mtb Δndh-2* from reductive stress stemming from the oxidation of long-chain fatty acids (9).

In addition to *ndh* and *ndhA*, the *Mtb* genome also contains the *nuo* operon, which encodes a nonessential type 1 NADH dehydrogenase (Ndh-1) (1). Unsurprisingly, Ndh-1 inhibitors do not restrict *Mtb* growth *in vitro*, even at high concentrations (1, 9). Nevertheless, Ndh-1 can compensate for the absence of Ndh-2 activity and support *Mtb* growth *in vitro* and in a mouse model of infection (9). *Mtb* lacking both the *nuo* operon and *ndh* is attenuated *in vivo*, suggesting that a mutant devoid of NADH dehydrogenase activity might not be viable (17). Consistent with this, the Ndh-1 inhibitor rotenone killed *Mtb Δndh-2 in vitro*, thus confirming Ndh-1 and Ndh-2 synthetic lethality (9). Here, we show that this synthetic lethality is due to impaired NADH oxidation. Moreover, we demonstrate that Ndh-1/Ndh-2 synthetic lethality can be achieved through chemical inhibition.

## RESULTS

### NADH dehydrogenase synthetic lethality is rescued by the heterologous expression of a NADH oxidase

**Fig 1:**
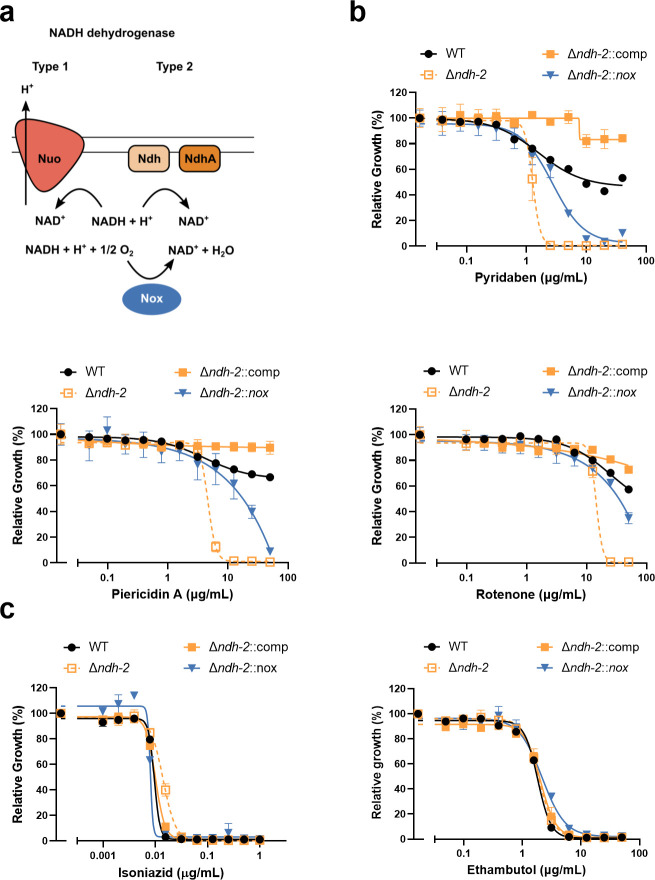
MIC measurements to test if expression of the water-forming NADH oxidase Nox (a) confers resistance to Ndh-1 inhibition in a *Δndh-2* genetic background. The wild-type (WT) strain, *Δndh-2* (*ΔndhΔndhA*) strain, complemented strain expressing one copy of *ndh* (*Δndh-2*::comp), and *Δndh-2* strain expressing *nox* (*Δndh-2::nox*) were grown in a modified Sauton’s minimal medium (fatty acid free; glucose and glycerol as carbon sources) and tested for susceptibility to the Ndh-1 inhibitors pyridaben, piericidin A, and rotenone (b) and to isoniazid and ethambutol as controls (c). Results correspond to OD_580nm_ normalized to the no-drug control on day 14 of treatment. Data are averages of technical triplicates. Error bars correspond to standard deviation. These data are representative of three independent experiments.

**Fig 2:**
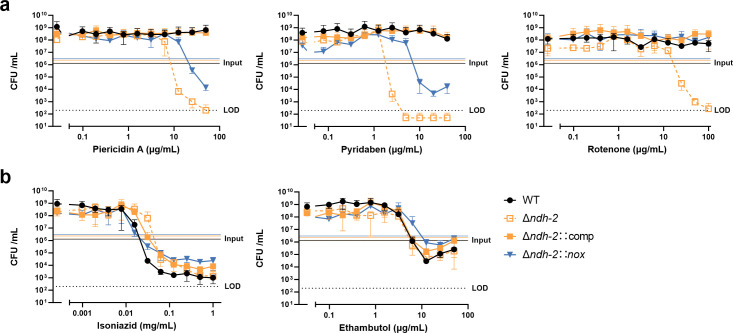
Bactericidal activity measurements. The WT strain, *Δndh-2* (*ΔndhΔndhA*) strain, complemented strain expressing one copy of *ndh* (*Δndh-2*::comp), and *Δndh-2* strain expressing *nox* (*Δndh-2::nox*) were grown in a modified Sauton’s minimal medium (fatty acid free; glucose and glycerol as carbon sources) and treated with the Ndh-1 inhibitors pyridaben, piericidin A, and rotenone (a) and isoniazid and ethambutol (b) for 14 days. Results correspond to colony-forming units (CFUs) per volume of culture (mL). Data are averages of technical triplicates. Error bars correspond to standard deviation. This experiment is representative of three independent experiments. LOD, limit of detection.

We have previously demonstrated that the chemical inhibition of Ndh-1 kills *Mtb Δndh-2* (9). In this work, we sought to understand the molecular mechanism of this synthetic lethality. NADH dehydrogenases play an important role in oxidative phosphorylation and NADH oxidation into NAD+. Previously, we showed that the heterologous expression of Nox*,* an enzyme that uses oxygen to convert NADH into NAD+ and water (Fig. 1a), rescued the sensitivity of *Mtb Δndh-2* to highly reduced carbon sources (9). We thus hypothesized that Nox could deconvolute the importance of NADH dehydrogenases in oxidative phosphorylation versus NADH oxidation. To test this, we determined the minimal inhibitory concentration (MIC) and minimal bactericidal concentration (MBC) of three different Ndh-1 inhibitors: pyridaben, piericidin A, and rotenone. As previously observed, the wild-type strain and complemented strain (*Δndh-2*::comp) were resistant to Ndh-1 inhibitors, while *Δndh-2* was hypersusceptible to all Ndh-1 inhibitors (9) (Fig. 1b). Interestingly, expression of *nox* in *Δndh-2* (*Δndh-2::nox*; Fig. S1) conferred increased resistance to all Ndh-1 inhibitors (Fig. 1b). This effect was not observed with isoniazid (INH) or ethambutol, showing that Nox does not confer unspecific resistance to antibiotics (Fig. 1c). Next, we measured bacterial viability upon treatment with the same panel of Ndh-1 inhibitors. As expected, all Ndh-1 inhibitors killed *Δndh-2* but not the wild-type and *Δndh-2*::comp strains (Fig. 2a). Expression of *nox* rescued *Mtb Δndh-2* from killing by rotenone and, to a lesser degree, pyridaben and piericidin A (Fig. 2a). The variability observed among different Ndh-1 inhibitors might be due to a saturation of Nox activity to overcome Ndh-1 inhibition and/or compound unspecific effects (Fig. 2a). This rescue effect was not observed in the control compounds INH and ethambutol (Fig. 2b). Interestingly, *Δndh-2* was less susceptible than the wild-type strain to INH (Fig. 2b), which is concordant with a previous report associating INH resistance with defective Ndh-2 activity in *Mycobacterium smegmatis* (18).

These data show that an alternative mechanism of NADH oxidation provided by Nox is capable of rescuing *Mtb* viability from a deficient NADH dehydrogenase activity. This observation indicates that the *Mtb* respiratory chain can compensate for the lack of functional NADH dehydrogenases, but the bacilli do not have an effective alternative mechanism for NADH oxidation.

### Inhibition of Ndh-1 in *Mtb Δndh-2* leads to depletion of intracellular NAD(H) pools

**Fig 3:**
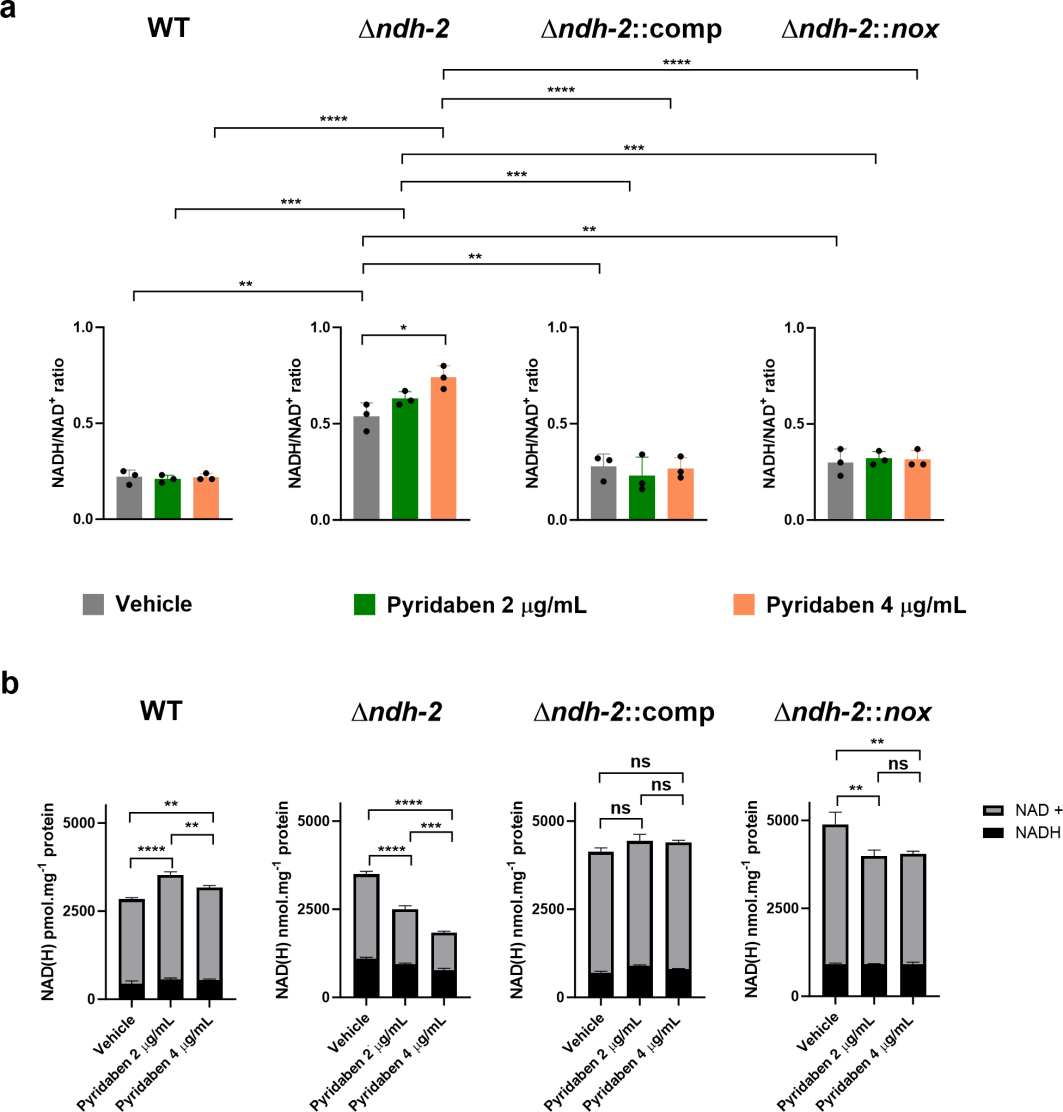
Intracellular NAD(H) concentrations. The WT strain, *Δndh-2* (*ΔndhΔndhA*) strain, complemented strain expressing one copy of *ndh* (*Δndh-2*::comp), and *Δndh-2* strain expressing *nox* (*Δndh-2::nox*) were grown in a modified Sauton’s minimal medium (fatty acid free; glucose and glycerol as carbon sources) until the mid-exponential phase (OD_580nm_ of 0.5) and treated with two concentrations of pyridaben (2 and 4 µg/mL) or vehicle (DMSO) for 24 h. (a) NADH/NAD+ ratio measurements are averages of three independent replicates. Error bars correspond to standard deviation. (b) NADH and NAD+ intracellular concentrations. Data are averages of three technical replicates. Error bars correspond to standard deviation. These data are representative of three independent experiments. Statistical significance was assessed by one-way ANOVA followed by a *post hoc* test (Tukey’s test; GraphPad Prism). **P  ≤ 0.01; ***P  ≤  0.001; ****P  ≤  0.0001. ns, not significant.

One of the possible consequences for an impairment in NADH oxidation is disruption of the cellular redox balance in the form of the NADH/NAD+ ratio. To test this hypothesis, we quantified intracellular NAD(H) pools in response to treatment with pyridaben (Pyr) at 2 and 4 µg/mL. We collected samples after 24 h of treatment to observe the primary effects of Ndh-1 inhibition in *Δndh-2* (growth arrest was only observed 48 h post-treatment; Fig. S2). As previously observed, *Δndh-2* displayed a higher NADH/NAD+ ratio than the wild-type and *Δndh-2*::comp strains in a medium compatible with growth (vehicle control) (9), which indicates that Ndh-2 has a more prominent role than Ndh-1 in redox homeostasis (Fig. 3a). Ndh-1 inhibition had a marginal but significant effect on the NADH/NAD+ ratio of *Mtb Δndh-2* (1.4× increase with the highest Pyr concentration). The wild-type, *Δndh-2*::comp, and *Δndh-2::nox* strains did not show significant alterations in the NADH/NAD+ ratio upon treatment with Pyr. The mild difference between the *Δndh-2* control (compatible with growth) and *Δndh-2* treated with Pyr (growth arrest) does not support the hypothesis of a deleterious disruption of the NADH/NAD+ ratio. However, *Δndh-2* treated with the highest concentration of Pyr also had an approximately threefold higher NADH/NAD+ ratio than the Pyr-treated wild-type strain. Thus, we cannot exclude that the observed increase in NADH/NAD+ contributes to Ndh-1/Ndh-2 synthetic lethality.

Next, we analyzed the individual intracellular concentrations of NADH and NAD+. Consistent with a defect in NADH oxidation, Pyr treatment depleted NAD+ (NADH dehydrogenase product) intracellular levels in *Δndh-2* in a dose-dependent manner (Fig. 3b). Curiously, Pyr treatment slightly lowered *Δndh-2* intracellular NADH (NADH dehydrogenase substrate) levels, which explains the mild increase in the NADH/NAD+ ratio. Inhibition of Ndh-1 in a *Δndh-2* genetic background thus leads to a decrease in the NAD(H) intracellular pool. The wild-type, *Δndh-2*::comp, and *Δndh-2::nox* intracellular NAD(H) levels did not show a Pyr dose-dependent effect.

Our results show that a defective NADH dehydrogenase activity leads to an increase in the NADH/NAD+ ratio and a depletion of NAD(H) intracellular pools, which is known to exert a bactericidal effect during infection (19, 20).

### Defective NADH dehydrogenase activity impacts oxidative phosphorylation indirectly

**Fig 4:**
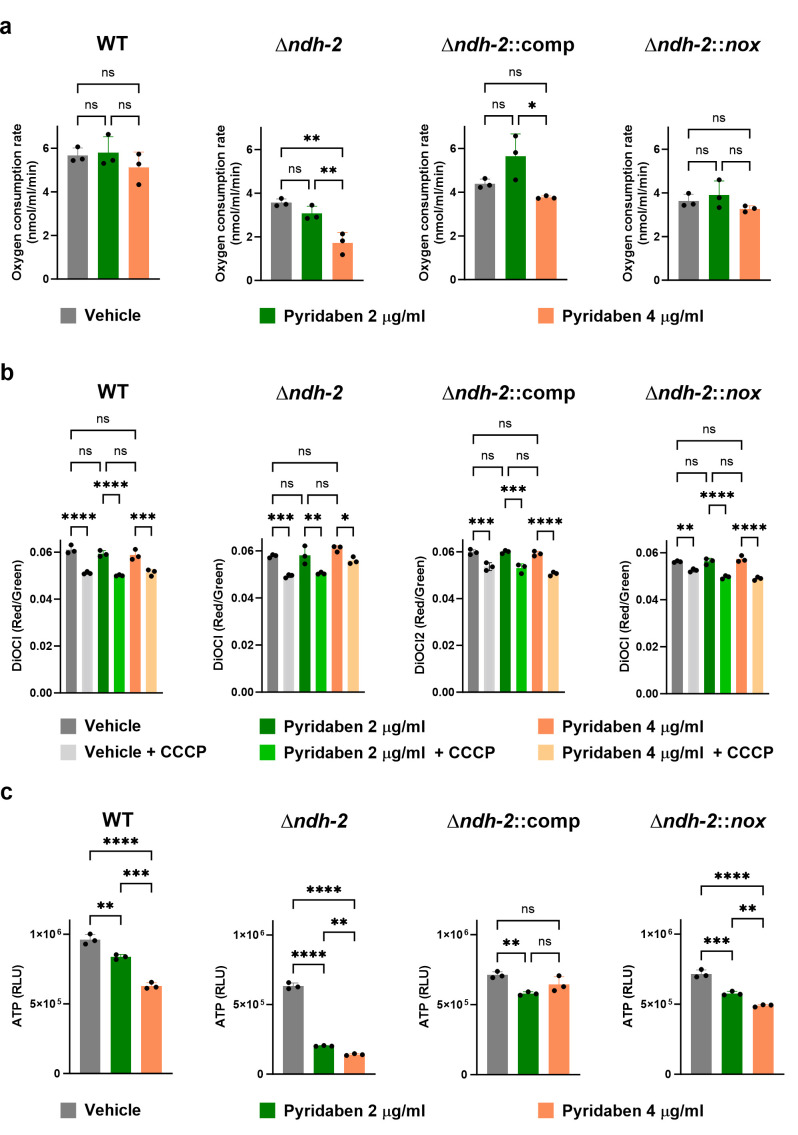
Quantification of oxidative phosphorylation-related variables in the WT strain, *Δndh-2* (*ΔndhΔndhA*) strain, complemented strain expressing one copy of *ndh* (*Δndh-2*::comp), and *Δndh-2* strain expressing *nox* (*Δndh-2::nox*). (**a**) Oxygen consumption rate (OCR) measurements. Glycerol (carbon source) and pyridaben (2 or 4 µg/mL) or vehicle (DMSO) were added to bacterial suspensions in PBS-tyloxapol (OD_580nm_ of 0.5) until a stable OCR was achieved. (**b**) Membrane potential measurement using the fluorescent probe DiOC_2_. Strains were cultured in a modified Sauton’s minimal medium (fatty acid free; glucose and glycerol as carbon sources) and treated with pyridaben (2 or 4 µg/mL) or vehicle (DMSO) for 24 h. Protonophore carbonyl-cyanide 3-chlorophenylhydrazone (CCCP) was used as a control for membrane depolarization. (**c**) Intracellular ATP levels. Strains were cultured in a modified Sauton’s medium (fatty acid free; glucose and glycerol as carbon sources) and treated with pyridaben (2 or 4 µg/mL) or vehicle (DMSO) for 24 h. Results are averages of technical triplicates. Error bars correspond to standard deviation. Data are representative of three independent experiments. Statistical significance was assessed by one-way ANOVA followed by a *post hoc* test (Tukey’s test; GraphPad Prism). *P  ≤ 0.05; **P  ≤ 0.01; ***P  ≤  0.001; ****P  ≤  0.0001. ns, not significant.

To assess if a defective overall NADH dehydrogenase activity compromises *Mtb* respiration, we measured the oxygen consumption rate (OCR) in bacterial suspensions treated with Pyr (2 and 4 µg/mL). In *Mtb Δndh-2,* Pyr treatment resulted in a concentration-dependent inhibition of oxygen consumption (Fig. 4a): ~15% inhibition with Pyr 2 µg/mL and ~48% inhibition with Pyr 4 µg/mL. The wild-type and *Δndh-2*::comp OCRs were insensitive to Pyr treatment. These results suggested a prominent role of NADH dehydrogenases in the input of electrons to the respiratory chain. However, *Δndh-2::nox* was also insensitive to Pyr treatment, indicating that the impact on respiration is indirect and stems from impaired NADH oxidation. Next, we measured membrane potential (Fig. 4b) and intracellular ATP levels (Fig. 4c) after a 24-h treatment with Pyr (2 and 4 µg/mL), using the same experimental design previously described (Fig. S2). This revealed that Pyr treatment did not depolarize the *Mtb Δndh-2* membrane. Nevertheless, Pyr lowered intracellular ATP levels in *Δndh-2*, which is consistent with a partial inhibition of oxygen respiration. The wild-type, *Δndh-2*::comp, and *Δndh-2::nox* strains showed only a modest but statistically significant effect on ATP intracellular levels. Of note, the OCRs and intracellular ATP levels of *Mtb Δndh-2* were lower than those of the wild-type strain in the vehicle control situation, which again argues in favor of Ndh-2 taking a more prominent role in *Mtb* physiology. Also, *Δndh-2*::comp did not show OCRs and intracellular ATP levels similar to those of the wild-type strain, which may be because this strain is only expressing *ndh*.

In summary, a defective overall NADH dehydrogenase activity negatively impacts *Mtb* oxidative phosphorylation. However, this stems from impaired NADH oxidation, which may inhibit multiple pathways necessary for a functional respiratory chain.

### Simultaneous chemical inhibition of Ndh-1 and Ndh-2 kills *Mycobacterium tuberculosis*

**Fig 5:**
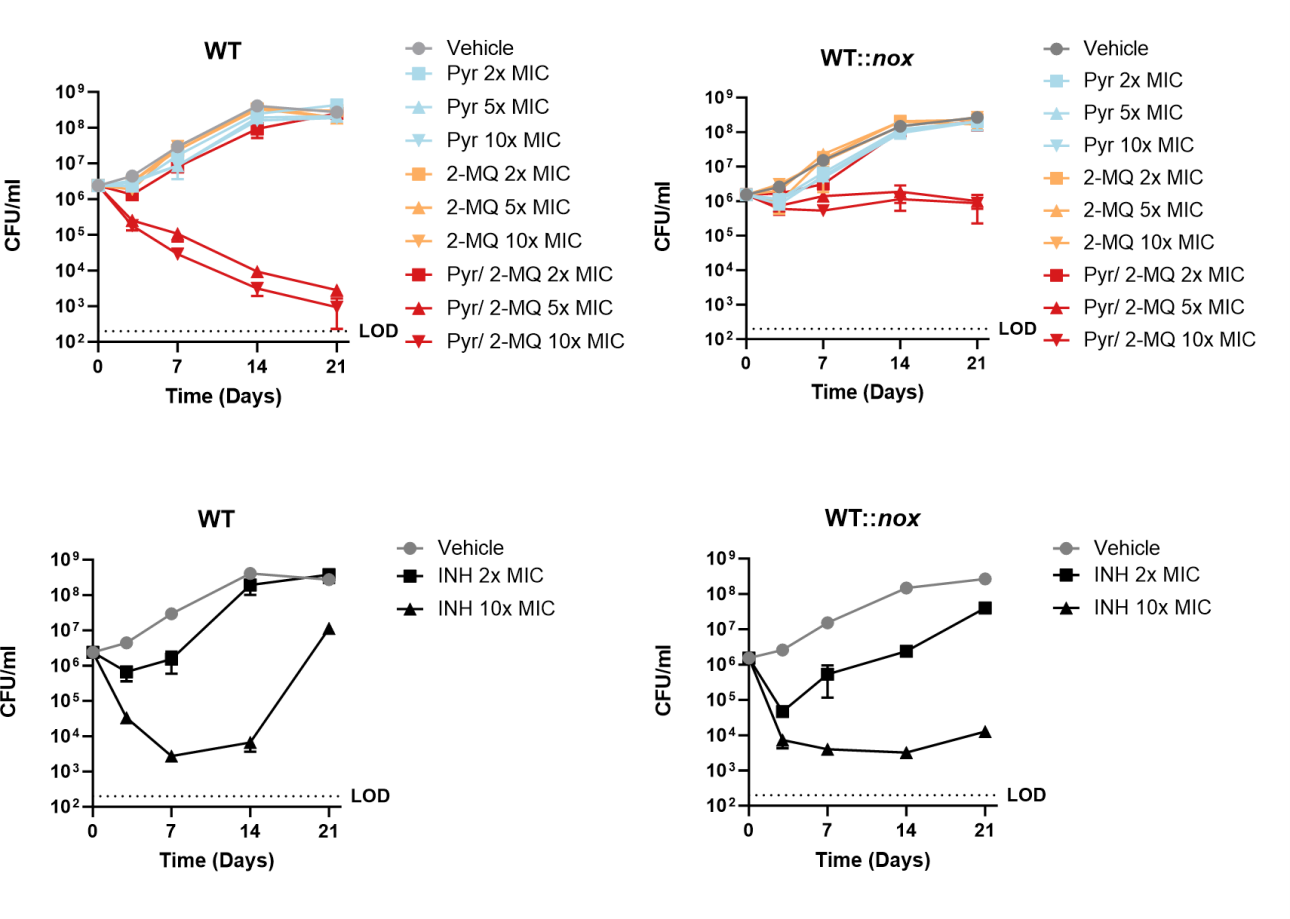
Kill curves. The wild-type (WT) strain and wild-type strain expressing *nox* (WT::*nox*) growing in a Sauton’s minimal medium (fatty acid free; glucose and glycerol as carbon sources) were treated with pyridaben (Pyr) or 2-mercapto-quinazolinone (2-MQ) DDD00853663 alone or in combination at concentrations (2×, 5×, or 10× MIC) based on Pyr MIC in *Δndh-2* or 2-MQ MIC in a modified Sauton’s medium. Isoniazid (INH) was used as a control at 2× and 10× MIC. Results are averages of technical triplicates. Error bars correspond to standard deviation. These data are representative of three independent experiments. LOD, limit of detection.

With the cytochrome bc1-aa3 oxidase inhibitor Q203 (Telacebec) (21) in clinical trials (22) and the discovery of a potent cytochrome bd oxidase inhibitor (23), synthetic lethality of *Mtb* terminal oxidases has been proposed as a new strategy to treat infections (24). We sought to verify if the same strategy could be applied to NADH dehydrogenases. To show that a bactericidal effect could be obtained through simultaneous chemical inhibition of both enzymes, we determined the killing kinetics of wild-type *Mtb* following treatment with the Ndh-1 inhibitor Pyr and the Ndh-2 inhibitor 2-Mercapto-Quinazolinone (2-MQ) DDD00853663 (14) individually or in combination (Fig. 5). Drug concentrations were based on MIC values for these compounds, which we determined in a previous study (9). To further support that defective NADH oxidation is the cause of synthetic lethality, we exposed wild-type *Mtb* expressing *nox* (Fig. S1) to the same conditions. INH was chosen as a control compound. The wild-type strain is not susceptible to Ndh-1 or Ndh-2 inhibition alone [Ndh-2 is only essential when long-chain fatty acids are present in the medium (9)]. However, simultaneous chemical inhibition of Ndh-1 and Ndh-2 led to a bactericidal effect. Consistent with our proposed mechanism, Nox rescued *Mtb* viability but not growth (Fig. S3) upon simultaneous treatment with Ndh-1 and Ndh-2 inhibitors. Importantly, this rescue effect was not observed with INH. To understand if the synthetic lethality observed with chemical inhibition led to similar physiological consequences as *Δndh-2* treated with Pyr, we measured the intracellular NAD(H) concentrations, OCRs, and intracellular ATP levels in the wild-type strain and wild-type strain expressing *nox* treated with Pyr, 2-MQ, and Pyr/2-MQ in combination (Fig. S4). Similar to *Δndh-2* treated with Pyr, the combination of Pyr and 2-MQ depleted the NAD(H) intracellular pool and decreased the OCR. Both effects were rescued by Nox. However, in the wild-type strain, we did not observe significant changes in the NADH/NAD+ ratio and detected only a mild negative effect on intracellular ATP levels, suggesting that these phenotypes are only observed when there is a complete absence of Ndh-2 activity.

We have also tested if treatment with a combination of Ndh-1 and Ndh-2 inhibitors kills *Mtb* in a non-replicative state using the PBST starvation model (Fig. S5). Our results show that in this model, simultaneous inhibition of Ndh-1 and Ndh-2 did not kill *Mtb*. Curiously, the strain expressing *nox* showed a loss of viability following treatment with both compounds alone and in combination. This effect was also observed, although to a lesser extent, in the vehicle control, indicating that Nox activity is deleterious during starvation.

Our results show that it is possible to exploit the synthetical lethality of *Mtb* NADH dehydrogenases through chemical inhibition, which leads to a depletion of NAD(H) intracellular pools and to an inhibition of respiration.

## DISCUSSION

The *Mtb* respiratory chain is a highly branched and plastic cellular process composed of nine respiratory dehydrogenases and four terminal oxidoreductases. The respiratory enzyme Ndh-2 was deemed to be required for *in vitro* growth (6, 7) and a major electron entry point to the respiratory chain (8). We have previously shown that Ndh-2 is conditionally essential for *in vitro* growth depending on the presence of highly reduced carbon sources and that Ndh-1 can compensate for the lack of Ndh-2 during mouse infection (9). Here, we sought to understand the molecular mechanism of Ndh-2/Ndh-1 synthetic lethality.

NADH dehydrogenases have two major functions: (i) NADH oxidation and (ii) input of electrons to the respiratory chain. We have previously shown that a water-forming NADH oxidase (Nox) is functional in *Mtb* (9) and reasoned that this enzymatic activity could disambiguate the roles of NADH dehydrogenases in NADH oxidation and respiration. We demonstrate that *nox* expression rescues *Mtb Δndh-2* (lacking *ndh* and *ndhA*) from the bactericidal effect of Ndh-1 chemical inhibition. NADH dehydrogenase activity is, thus, essential for bacilli viability, at least in the tested conditions, due to its role in NADH oxidation rather than being the major donor of electrons to the respiratory chain.

The measurement of intracellular NAD(H) pools in *Δndh-2* challenged with chemical Ndh-1 inhibition showed a depletion of NAD+, which is consistent with impaired NADH oxidation. However, depletion of NAD+ was not accompanied by the accumulation of NADH. On the contrary, intracellular NADH levels decreased in response to Pyr treatment, resulting in only a modest increase in the NADH/NAD+ ratio. This is in contrast with what was observed in other bacteria, where NADH dehydrogenases are essential for NADH/NAD+ homeostasis (25). The facultative anaerobic pathogen *Listeria monocytogenes* is a more extreme case of this, as it was shown to use oxygen respiration primarily to oxidize NADH and balance NADH/NAD+ (26). Instead, Ndh-1 chemical inhibition in *Δndh-2* led to a decrease in the intracellular NAD(H) pools, an effect that was shown to be bactericidal to *Mtb in vitro* and during infection (20). Importantly, Nox restored the NAD(H) intracellular concentrations of *Mtb Δndh-2* to wild-type levels. These phenotypes are thus consistent with a model in which the absence of an efficient NADH oxidation system triggers a mechanism that avoids NADH accumulation to balance the NADH/NAD+ ratio; however, in doing so, it traps the bacilli in a feedback loop that leads to NAD(H) intracellular pool depletion. The nature of this mechanism is unknown, but one possibility could be the NADH-inducible nudix hydrolase RenU that cleaves NADH with high specificity (27).

The evaluation of bioenergetic parameters, namely, the OCR, membrane potential, and intracellular ATP levels, was consistent with a negative impact of deficient overall NADH dehydrogenase activity in oxidative phosphorylation. However, the fact that these phenotypes were complemented by the expression of *nox* argues in favor of an indirect effect stemming from impaired NADH oxidation. *Mtb* expresses multiple dehydrogenases capable of reducing menaquinone, including succinate dehydrogenases that were recently shown to be essential for growth (28), which may compensate for the lack of NADH dehydrogenase activity. This is in contrast with other bacterial pathogens like *Streptococcus agalactiae*—a causal agent of sepsis in newborns and immunocompromised adults—which uses NADH dehydrogenases as the main entry point of electrons to the respiratory chain (29).

Treatment of *Mtb* with a combination of Ndh-1 and Ndh-2 inhibitors showed that a bactericidal effect can be achieved by chemical inhibition. Importantly, expression of *nox* rescued wild-type *Mtb* from Ndh-1/Ndh-2 synthetic lethality, further confirming that NADH oxidation is the main biological function of NADH dehydrogenases regarding bacterial viability. As observed through chemical genetics, simultaneous Ndh-1 and Ndh-2 inhibition led to a decrease in the intracellular NAD(H) pools and a decrease in OCRs. These effects were counteracted by *nox* expression. On the contrary, NADH/NAD+ and ATP intracellular levels did not behave as observed in *Δndh-2* treated with Pyr. Thus, the synthetic lethality obtained with the combination of Ndh-1 and Ndh-2 inhibitors is likely a result of depleted NAD(H) intracellular pools (19, 20) and, possibly, an effect of respiration inhibition independent of ATP depletion. During PBS starvation, simultaneous Ndh-1 and Ndh-2 chemical inhibition did not kill *Mtb*, showing that this inhibitor combination is not effective in some non-replicative environments. This contrasts with hypoxia, where *Mtb* is also in a non-replicative state and the absence of Ndh-2 alone kills *Mtb* (9). The mechanism behind the protective effect of starvation over Ndh-1/Ndh-2 simultaneous inhibition is unknown and merits further investigation. Interestingly, *nox* expression sensitized *Mtb* to both inhibitors alone and in combination. A decline in CFUs was also observed in the vehicle control. *Mtb* is known to increase NADH/NAD+ when in a non-replicative state (30); thus, it is possible that Nox may be counteracting this adaptive process.

Several potent Ndh-2 inhibitors have been identified (10–16). *Mtb Δndh-2 in vitro* susceptibility to long-chain fatty acids (9), an important carbon source during infection, and to hypoxic conditions (9) argues for Ndh-2 inhibitors to be effective in specific microenvironments like the necrotic center of caseating granulomas. However, the fact that *Mtb Δndh-2* grows and survives during mouse infection (9) shows that Ndh-2 inhibition may not be effective in all infection microenvironments. To potentiate Ndh-2 inhibitors, Ndh-1/Ndh-2 synthetic lethality could be exploited. The main challenge to this approach would be the identification of inhibitors with high affinity for *Mtb* Ndh-1 and low affinity for the homologous enzyme in humans (complex I). The currently known Ndh-1 inhibitors interact with the quinone-binding pocket (31) of species across the tree of life, including humans, making them toxic. However, one could make a case for such an endeavor given the successful identification of inhibitors to *Mtb* enzymes that have homologs in humans, like bedaquiline (2) (ATP synthase) and Q203 (21) (cytochrome *bc1* complex). Moreover, the structure of the mycobacterial Ndh-1 enzyme complex was recently solved (32), which could help in the design of Ndh-1 inhibitors.

Our work unveiled NADH oxidation as the essential function of NADH dehydrogenases for maintaining *Mtb* viability. Deficient overall NADH dehydrogenase activity was associated with depletion of NAD(H) intracellular tools and inhibition of respiration. Finally, we showed that Ndh-1/Ndh-2 synthetic lethality can be exploited to kill *Mtb* by chemically inactivating these enzymes.

## MATERIALS AND METHODS

### Growth conditions and strains

*Mtb* strains (H37Rv genetic background) were cultured in a modified Sauton’s minimal medium: 0.05% (wt/vol) potassium dihydrogen phosphate, 0.05% (wt/vol) magnesium sulfate heptahydrate, 0.2% (wt/vol) citric acid, 0.005% (wt/vol) ferric ammonium citrate, and 0.0001% (wt/vol) zinc sulfate, supplemented with 0.05% (vol/vol) tyloxapol, 0.4% (wt/vol) glucose, 0.2% (vol/vol) glycerol, and ADNaCl with fatty acid-free BSA (Roche). For the modified Sauton’s solid medium, 1.5% (wt/vol) bactoagar (BD) was added; also, glycerol was added at 0.5% (wt/vol). Solid medium was used for transformation and CFU outgrowth. When necessary, antibiotics were added to cultures (final concentrations): hygromycin 50 µg/mL, kanamycin 25 µg/mL, streptomycin 50 µg/mL, and zeocin 12.5 µg/mL. *Δndh-2*, *Δndh-2* complemented (expression of a native copy of *ndh*), and *Δndh-2::nox* (strain with an integrative plasmid expressing an *Mtb* codon-adapted version of *nox* from *Lactococcus lactis* under the transcriptional control of the promoter Ptb38—pGMCgS-0×-Ptb38-NOX-FLAG-SD1) were generated in a previous study (9). For this study, we have transformed the wild-type strain with the integrative plasmid pGMCgS-0×-Ptb38-NOX-FLAG-SD1 to generate WT::*nox*.

### Immunodetection

WT, Δ*ndh-2*, Δ*ndh-2*::comp, Δ*ndh-2::nox,* and WT::*nox* cultures were grown until the mid-exponential phase. Bacteria were washed with PBS with 0.05% tyloxapol and resuspended in 500  µL of PBS with 1× protease inhibitor cocktail (Roche). Bacterial lysis was performed by bead-beating three times at 4,500  rpm for 60  s with 0.1-mm zirconia/silica beads. Supernatants were then filtered through a 0.2-µm SpinX column (Corning). Protein concentrations were determined using a Qubit Protein Assay Kit (Invitrogen). We used 30  µg of protein, separated the protein extracts through SDS-PAGE, transferred them to nitrocellulose membranes, and proceeded with the incubation with primary antibodies, anti-Flag (Sigma-Aldrich, at 1∶1,000 dilution) and anti-PrcB (Sigma-Aldrich, at 1∶1,000 dilution), at room temperature for 2 h. The secondary antibodies, goat anti-mouse IgG (Thermo Fisher, DyLight 800) and donkey anti-rabbit IgG (LI-COR Biosciences, IRDye 680LT), were used at a 1:10,000 dilution and incubated at room temperature for 30 min. Immunodetection was performed in an Odyssey Infrared Imaging System (LI-COR Biosciences).

### Minimal inhibitory/bactericidal concentration and kill curves

For MIC assays, strains were grown in the modified Sauton’s medium until the mid-exponential phase (OD_580nm_ of 1) and resuspended in a fresh medium as single bacterial suspensions. Ninety-six-well plates with 11 concentration drugs (twofold dilutions) plus a no-drug control in triplicate (dispensed by D300e Digital Dispenser, HP) were seeded with 200 µL of single bacterial suspensions. DMSO was normalized across wells to 1% (vol/vol). The plates were incubated for 14 days, and OD_580nm_ was then recorded. Results were presented as a percentage of drug over no-drug control. To estimate MBC, we took samples from the MIC plates and serially diluted the culture in PBS-tyloxapol 0.05% (vol/vol). Modified Sauton’s solid medium was used for outgrowth. Kill curves were performed in 96-well plates following the same culture conditions described for MIC/MBC. Samples for CFU determination were harvested on days 3, 7, 14, and 21. For kill curves in starvation conditions, strains were grown in modified Sauton’s medium until the exponential phase (OD_580nm_ of 0.6–2.0) and washed twice in PBS buffer with 0.05% tyloxapol (PBST) and resuspended in PBST to a final OD_580nm_ of 0.01. Bacteria were starved for 7 days and then seeded in 96-well plates and treated with the following drug concentrations: 2-MQ, 1.5 and 3 µg/mL; pyridaben, 12.5 and 25 µg/mL; rifampicin, 0.5 and 1 µg/mL; or vehicle (DMSO). CFUs were enumerated by culturing serial dilutions on modified Sauton’s solid medium supplemented with charcoal 0.4% (wt/vol) before drug exposure and at 1, 7, 14, 21, and 28 days post-treatment. Drugs were dispensed by a D300e Digital Dispenser (HP), with DMSO being normalized to 1% (vol/vol) across wells. Modified Sauton’s solid medium was used for outgrowth.

### NAD(H) quantification, membrane potential, and intracellular ATP levels

NAD(H) quantification, membrane potential, and ATP intracellular levels were determined using the same experimental design. *Mtb* strains were cultured (10 mL; unvented T-75 flasks; with rotation 100 rpm) in modified Sauton’s minimal medium until the mid-exponential phase (OD_580nm_ of 1) and resuspended in fresh medium at an OD_580nm_ of 0.5; DMSO and pyridaben (2 and 4 µg/mL) were then added to the cultures (DMSO was normalized to the highest concentration across conditions). Bacteria were harvested after a 24-h treatment (time point that precedes the inflection point of *Δndh-2* growth treated with pyridaben). NAD(H) intracellular concentration was quantified with the commercial kit Fluoro NAD (Cell Technology) following the manufacturer’s instructions. NAD(H) intracellular concentrations were normalized by protein content (Qubit Protein Assay Kit, Invitrogen). For membrane potential, bacteria were resuspended in fresh medium (same experimental conditions) with a final OD_580nm_ of 1 and treated with 15 µM DiOC_2_ for 30 min (room temperature). Protonophore carbonyl-cyanide 3-chlorophenylhydrazone (CCCP) was added at a final concentration of 50 µM to provide a depolarized membrane control. Samples were then washed in fresh media (same experimental conditions), and 200 µL in triplicate was dispensed in black clear bottom 96-well plates (Costar). Fluorescence was recorded in a SpectraMax M5 spectrofluorimeter (Molecular Devices): green fluorescence (488 nm/530 nm) and red fluorescence (488 nm/610 nm). A shift to red is synonymous with dye aggregation caused by membrane potential. Membrane potential was estimated as a ratio of red fluorescence over green fluorescence. ATP intracellular levels were determined using the commercial kit BacTiter-Glo (Promega) following the manufacturer’s instructions.

### Oxygen consumption rate quantification

*Mtb* strains were cultured in modified Sauton’s medium until the mid-exponential phase (OD_580nm_ of 1) and resuspended in pre-heated (37°C) PBS-tyloxapol 0.05% (vol/vol) to a final OD_580nm_ of 0.5. A Clark-type electrode system (Oxytherm+, Hansatech) was used to measure dissolved oxygen concentration. After calibrating the instrument following the manufacturer’s instructions, we added the following components to the chamber: 950 µL of bacterial suspension, 25 µL of glycerol (1 M), and 25 µL of pyridaben (40× treatment concentration) or DMSO (vehicle). The oxygen concentration was followed and recorded using the software Oxytrace+.

## SUPPLEMENTAL MATERIAL

Supplemental Figures mbio.01045-23-s0001.pdf Fig. S1 to S5.

ASM does not own the copyrights to Supplemental Material that may be linked to, or accessed through, an article. The authors have granted ASM a non-exclusive, world-wide license to publish the Supplemental Material files. Please contact the corresponding author directly for reuse.
