# Supplementary material for: Synthetic lethality of Mycobacterium tuberculosis NADH dehydrogenases is due to impaired NADH oxidation
Source: mBio. 2023 Nov 30;14(6):e01045-23. doi: 10.1128/mbio.01045-23 (PMC10746327; doi:10.1128/mbio.01045-23)
Supplement: Supplemental Figures — Fig. S1 to S5. [file mbio.01045-23-s0001.pdf]

Supplementary Information

Mycobacterium tuberculosis type 1 and type 2 NADH dehydrogenase synthetic lethality is due to impaired NADH re-oxidation

Yuanyuan Xu, Sabine Ehrt, Dirk Schnappinger, Tiago Beites

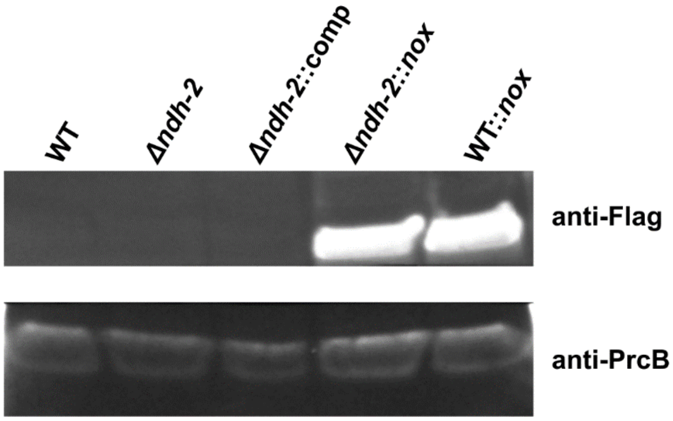

FIG S1 Immunodetection of Nox-Flag expression. Nox-Flag was detected with an anti-flag antibody. PrcB was used as a loading control.

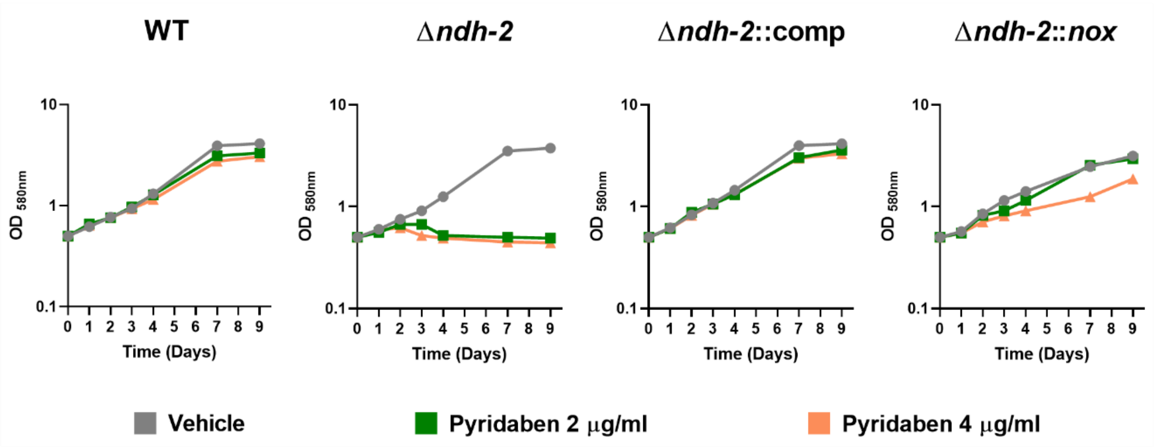

FIG S2 Growth kinetics upon treatment with Pyridaben (2 μg/ml and 4 μg/ml) or vehicle DMSO. Drug was added at day 1, and samples were harvested at day 2. These results are representative of 5 independent experiments.

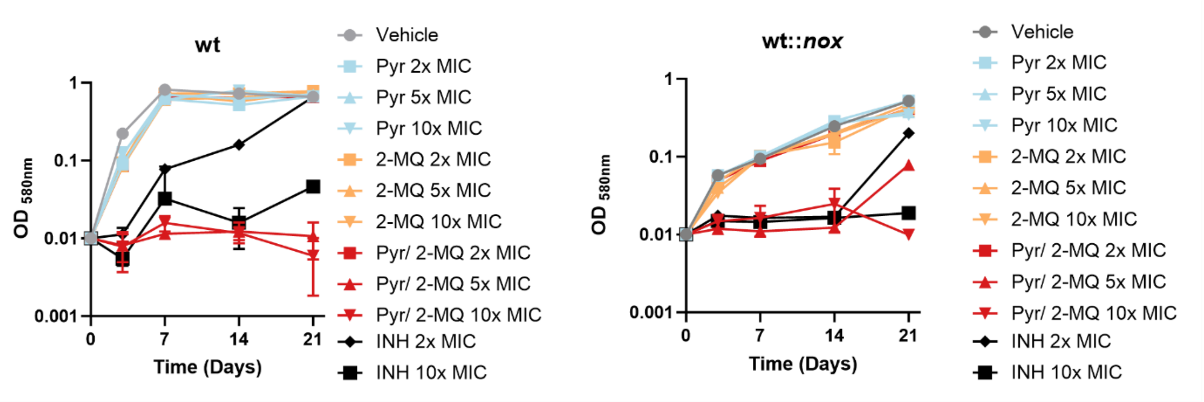

FIG S3 Growth kinetics of wild-type (WT) and wild-type expressing *nox* (WT::*nox*) treated with pyridaben (Pyr) or the 2-Mercapto-Quinazolinone (2-MQ) DDD00853663 alone or in combination at concentrations (2x, 5x or 10x MIC). Isoniazid (INH) at 2x and 5x MIC was used as a positive control. Strains were grown in Sauton's minimal medium (fatty acid free; glucose and glycerol as carbon sources). Results are averages of technical triplicates. Error bars correspond to standard deviation. These data are representative of 3 independent experiments.

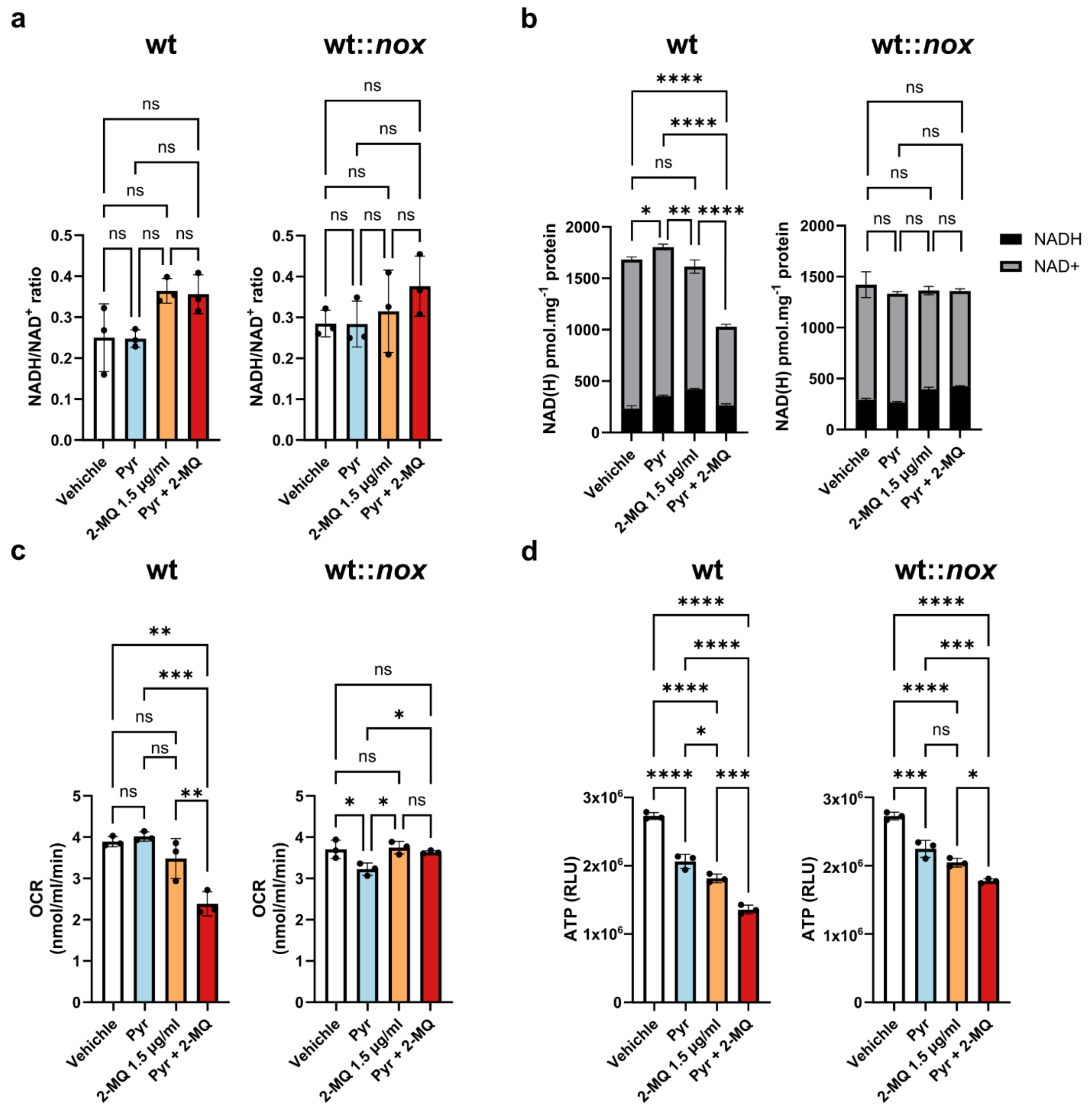

FIG. S4 Physiological characterization of wild-type (WT) and wild-type expressing *nox* (WT::*nox*) in response to pyridaben (Pyr) 5x MIC, 2-Mercapto-Quinazolinone (2-MQ) DDD00853663 5x MIC and both drugs in combination. Strains were cultured in modified Sauton's (fatty acid free; glucose and glycerol as carbon sources). (a) NADH/NAD+ ratio and (b) intracellular NAD(H) concentration in response to a 24 h treatment. (c) Oxygen consumption rate (OCR). Glycerol (carbon source) and Pyr, 2-MQ, a combination of both compounds, or vehicle (DMSO) were added to bacteria suspensions (from a culture in logarithmic phase) in PBS-tyloxapol (OD_{580nm} of 0.5) until a stable OCR was achieved. (d) ATP intracellular levels in response to a 24 h treatment. Results are averages of technical replicates. Error bars correspond to standard deviation. Data are representative of 3 independent experiments. Statistical significance was assessed by one-way

ANOVA followed by post hoc test (Tukey test; GraphPad Prism). *P ≤ 0.05; **P ≤ 0.01; ***P ≤ 0.001; ****P ≤ 0.0001; ns - not significant.

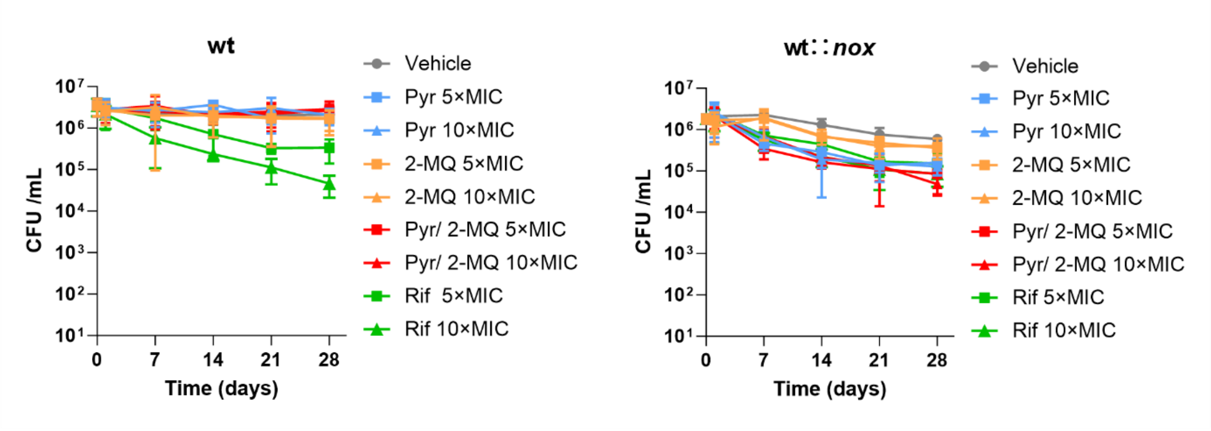

FIG. S5 Survival of wild-type (WT) and wild-type expressing *nox* (WT::*nox*) to treatment with pyridaben (Pyr) 5x and 10x MIC, 2-Mercapto-Quinazolinone (2-MQ) DDD00853663 5x and 10 MIC or in combination during PBS-tyloxapol starvation. Results are averages of technical triplicates. Error bars correspond to standard deviation. These data are representative of 2 independent experiments.
